# German mammography screening program: program sensitivity between 2010 and 2016 estimated based on German health claims data

**DOI:** 10.1186/s12885-023-11378-0

**Published:** 2023-09-11

**Authors:** Franziska Heinze, Jonas Czwikla, Miriam Heinig, Ingo Langner, Ulrike Haug

**Affiliations:** 1 Department of Health, Long-Term Care and Pensions, SOCIUM Research Center on Inequality and Social Policy, University of Bremen, Mary-Somerville-Straße 5, Bremen, 28359 Germany; 2 Department of Clinical Epidemiology, Leibniz Institute for Prevention Research and Epidemiology - BIPS, Achterstraße 30, Bremen, 28359 Germany; 3 High-Profile Area of Health Sciences, University of Bremen, Bibliothekstraße 1, Bremen, 28359 Germany

**Keywords:** Breast cancer, Mammography, Claims data, Screening

## Abstract

**Background:**

Program sensitivity is a key quality indicator for mammography screening programs (MSP). Estimating program sensitivity usually requires a linkage of screening and cancer registry data. For the German MSP, such data linkage-based estimates have only been reported for two out of 16 federal states. We aimed to explore the potential of estimating program sensitivity for the German MSP based on information available in health claims data.

**Methods:**

We used data from the second-largest statutory health insurance fund in Germany, BARMER (~ 9 million members all over Germany). We included women aged 50 to 69 years with a non-initial screening mammography between 2010 and 2016 and followed them up for two years. We estimated the rate of screen-detected and interval cancers as well as program sensitivity.

**Results:**

Per year, we included 212,400 to 303,667 women (mean age: 60–61 years). Overall, 1,992,287 non-initial MSP screening examinations conducted in these women between 2010 and 2016 were considered for the analyses. Age-standardized program sensitivity ranged between 69.9% [95% CI: 67.3–72.0%] and 71.7% [95% CI: 69.5-73.9%] during the study period. Per 1,000 non-initial screening examinations, the rate of screen-detected breast cancer ranged between 4.6 and 5.3, and the rate of interval breast cancer rates ranged between 0.6 and 0.8 for the first and between 1.3 and 1.4 for the second year after screening.

**Conclusions:**

Our results were plausible and consistent with quality indicators estimated for the German MSP based on data linkage and thus support the value of German health claims data in this regard. The quality indicators estimated in our study are in line with levels expected according to European Guidelines.

**Supplementary Information:**

The online version contains supplementary material available at 10.1186/s12885-023-11378-0.

## Background

In 2018, breast cancer accounted for almost one third of new cancer cases (N = 69.900) and was the leading cause of cancer death (N = 18.591 of 104.791 cancer deaths) among women in Germany [1]. In Germany, an organized Mammography Screening Program (MSP), inviting women aged 50 to 69 years to a mammography every two years, was stepwise introduced between 2005 and 2008 and has reached full coverage since 2009.

Following the European guidelines for quality assurance in breast cancer screening and diagnosis [2], several parameters are defined in the German MSP to assess its quality and to indirectly estimate the effectiveness of the program. A key quality indicator is program sensitivity, defined as the percentage of new breast cancers detected within the MSP (i.e. screen-detected breast cancers) divided by the total of screen-detected and interval breast cancers. Interval breast cancers are typically defined as breast cancers diagnosed in the interval between a negative mammography and the next regular screening procedure.

While the number of screen-detected cancers is directly available from the program documentation, determining the number of interval cancers is more complicated. It is usually ascertained based on a data linkage between cancer registry data and adherence data from the MSP. In Germany, however, data linkage is challenging due to strict data protection regulations. Furthermore, the fact that there are 11 different epidemiological cancer registries in Germany with differences in the legal basis and in data processing hampers determining these parameters at a national level. To date, interval cancer rates and program sensitivity have only been reported for two federal states (Lower Saxony and North Rhine-Westphalia (NRW)) for the years 2005 to 2011 [3–5]. Estimating these parameters based on statutory health insurance claims data might be an additional approach worth exploring given that claims data are readily available and include codes for breast cancer diagnoses, mammography screening as well as further diagnostic workup and treatment.

We, therefore, aimed (I) to estimate the rates of screen-detected and interval breast cancers and – by combining both – the program sensitivity for the German MSP based on data from a large statutory health insurance fund and (II) to compare the estimates with previously published results generated based on data linkage by Bokhof et al. [3].

## Methods

### Data source

We used claims data of the second-largest statutory health insurance fund in Germany, BARMER. With almost 9 million members all over Germany, the data cover about 12% of the German statutorily (non-private) health-insured population. About 90% of the German population is a member of the statutory health insurance [6, 7]. The claims data contain i.a. information on in- and outpatient diagnoses and medical procedures and services according to the German uniform assessment standard (EBM) and the Operations and Procedures Coding System (OPS) as well as demographic information. Diagnoses are coded according to the German Modification of the International Classification of Diseases, 10th Revision (ICD-10-GM). Whereas exact dates are predominantly available for inpatient diagnoses, outpatient diagnoses are transmitted to the statutory health insurance on a quarterly basis. For outpatient diagnoses, the additional coding of diagnostic certainty is mandatory, differentiating between “certain“, “suspected”, “status post”, and “ruled out”. Procedures related to the MSP have specific codes and can thus be distinguished from diagnostic mammographies and follow-up diagnostics. For this study, we used data from 2007 to 2019.

### Study design and study population

We included women aged 50 to 69 years with a non-initial screening mammography (EBM 01750) between 2010 and 2016 and with unambiguous information on sex, year of birth, and place of residence. Non-initial screening mammography was defined as any screening mammography that occurred after a prior screening mammography during the pre-observation. The quarter before this non-initial screening mammography was defined as the index quarter (cohort entry). This definition allowed us to also consider relevant procedures erroneously coded before the screening mammography (this was observed in patient profile reviews, even though not often). The pre-observation period was defined as the 11 quarters before the index quarter. We excluded women who were not continuously insured during this pre-observation period. We focused on non-initial screening mammographies as it is known that performance indicators differ widely between initial and non-initial mammographies [2]. If multiple non-initial screening mammographies were coded in the same woman within one quarter, we considered it as only one mammography; mammographies coded in the same woman but in different quarters of a year were considered separately for the analyses.

After cohort entry, included women were followed up for 9 quarters (index quarter, quarter of screening mammography and 7 additional quarters), their next MSP screening or death, whichever occurred first. As outpatient diagnoses and outpatient OPS codes in claims data are only available quarterly, we considered the occurrence of diagnoses and procedures only on a quarterly basis.

### Identification of breast cancer cases

To identify invasive (ICD C50) and in-situ (ICD D05) breast cancers we considered diagnosis codes from the in- and outpatient setting. To be classified as an incident case (invasive breast cancer or carcinoma in situ) the diagnosis had to be (a) coded as an inpatient primary discharge diagnosis or coded in two consecutive quarters as either (b) twice in the outpatient setting (first diagnosis labelled “certain” and second diagnosis labelled “certain” or “status post”) or (c) coded in both settings (initially with an outpatient diagnosis labelled “certain” and secondly with an inpatient primary or ancillary hospital diagnosis). The first quarter during the follow-up period in which the first of these diagnoses occurred was defined as the quarter of diagnosis. To distinguish between incident and prevalent cases, we also assessed whether there was already an inpatient code or an outpatient code labelled as “certain” or “status post” for invasive or in situ breast carcinoma recorded during the pre-observation period. If this was the case, the cancer was defined as a prevalent case and thus not considered in the calculations of screen-detected / interval cancer rates or program sensitivity.

### Classification of incident breast cancers into screen-detected vs. interval cancer

**Fig. 1:**
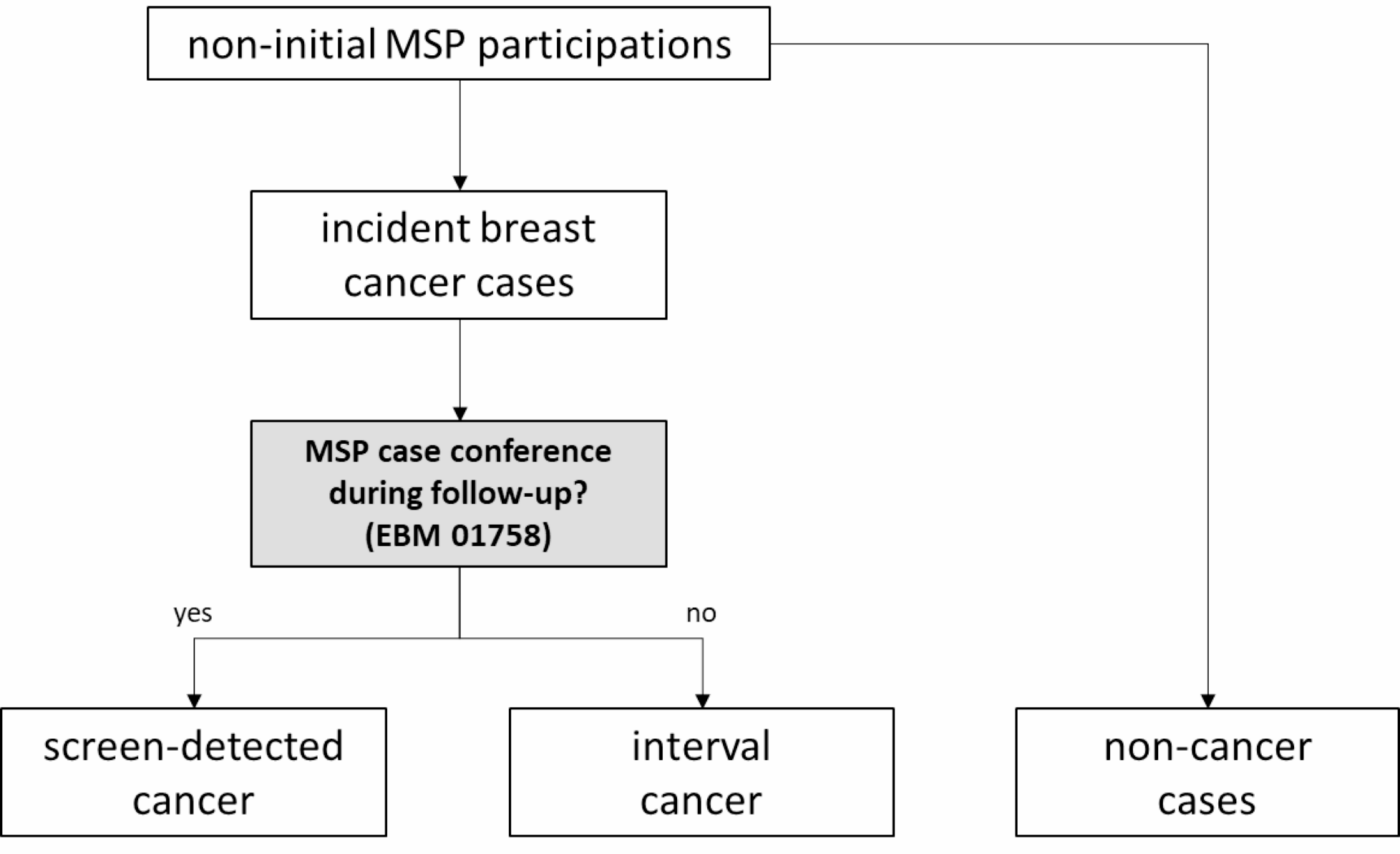
Algorithm for the distinction between screen-detected and interval cancers with health claims data

To classify incident breast cancers into screen-detected vs. interval cancer we established an algorithm illustrated in Fig. 1 [8]. It started with considering all women with a non-initial MSP examination in the respective year fulfilling the inclusion criteria (in case a woman had unexpectedly two non-initial MSP examinations per year, she was counted twice). For all included women, we assessed whether there was an incident breast cancer diagnosis (invasive or in-situ) as defined above during follow-up. To classify these cases into screen-detected vs. interval cancer, the code for a multidisciplinary pre-operative case conference after suspicious findings at screening mammography (EBM 01758) was a key indicator. Such a case conference is supposed to be held for all suspicious findings where a biopsy was taken in the context of the MSP. We thus defined all incident breast cancer cases with at least one code for such a case conference during the follow-up period as screen-detected. All other incident breast cancer diagnoses were defined as interval cancers.

### Data analysis

We calculated “rates” (in a statistical sense: proportions) for screen-detected and interval breast cancers as well as program sensitivity for each screening year between 2010 and 2016. All analyses were conducted nationwide and - to facilitate comparison with previously published data - for the biggest German federal state, i.e. North Rhine-Westphalia, separately. Due to a smaller sample size, we decided to forgo separate analyses for Lower Saxony. The rate of interval cancer was calculated as the proportion of women with non-initial MSP participation in the respective year who developed interval cancer among all non-initial MSP examinations in the respective year (expressed per 1,000). In additional analyses, we differentiated between interval cancers occurring in the first vs. the second year after non-initial MSP participation. The rate of screen-detected breast cancer was calculated as the proportion of women with non-initial MSP participation in the respective year in whom breast cancer was detected at the MSP among all non-initial MSP examinations in the respective year (expressed per 1,000). Program sensitivity was calculated as the number of screen-detected cancers divided by the sum of screen-detected and interval cancers. 95% confidence intervals were calculated for all indicators [9]. All indicators were age-standardized using the old standard population of Europe as reference in line with Bokhof et al. [3].

## Results

Overall, 1,992,287 non-initial MSP screening examinations (of which 531,037 in North Rhine-Westphalia) were included between 2010 and 2016. Table 1 shows—for each year—the number of women in whom these screening examinations were conducted, their age distribution, and the mean follow-up time. Per year we included 212,400 to 303,667 women. Their mean age ranged between 60.1 (SD 4.8) in 2014 and 60.6 in 2010 (SD 5.2). Age distribution stayed nearly constant between 2010 and 2016, thus barely influencing (non)-standardized time trends. The mean time period between non-initial MSP screening examination and end of follow-up ranged between 1.97 and 1.98 years (SD 0.08), i.e. follow-up was complete for almost all women.

**Table 1:** Characterization of the study population for each year between 2010 and 2016

	2010		2011		2012		2013		2014		2015		2016	
	N	%	N	%	N	%	N	%	N	%	N	%	N	%
Number of women (N)	212,400		276,135		298,843		303,667		302,568		299,404		299,170	
Age (years)														
50–54	33,095	15.6	45,316	16.4	45,941	15.4	49,365	16.3	49,558	16.4	49,526	16.5	48,136	16.1
55–59	60,836	28.6	80,141	29.0	90,367	30.2	91,648	30.2	91,593	30.3	90,419	30.2	89,385	29.9
60–64	56,985	26.8	78,394	28.4	87,265	29.2	89,173	29.4	88,297	29.2	84,514	28.2	83,996	28.1
65–69	61,484	28.9	72,284	26.2	75,270	25.2	73,481	24.2	73,120	24.2	74,945	25.0	77,653	26.0
mean (SD)	60.6 (5.2)		60.4 (5.1)		60.3 (5.0)		60.2 (5.0)		60.1 (4.9)		60.1 (5.0)		60.3 (5.0)	
Number of non-initial screening examinations (N)	212,408		276,143		298,852		303,685		302,590		299,423		299,186	
Mean time of follow-up (SD)	1.97 (0.08)		1.98 (0.08)		1.98 (0.08)		1.98 (0.08)		1.98 (0.08)		1.97 (0.08)		1.97 (0.08)	

### Results for North Rhine-Westphalia and comparison with external data source

**Fig. 2:**
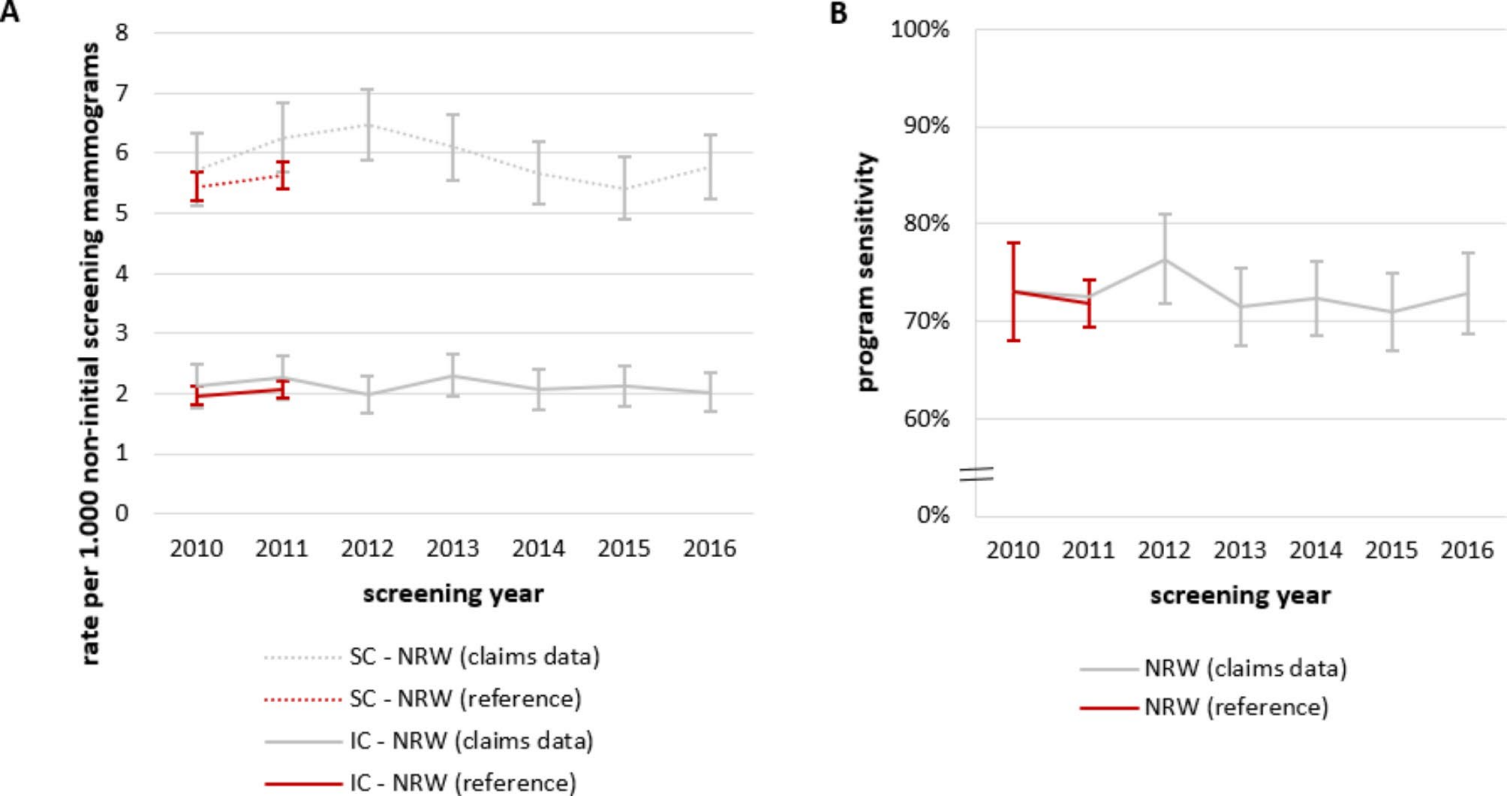
(**A**) Rates of screen-detected (SC) and interval cancers per 1,000 non-initial screening examinations estimated based on claims data compared to data published by Bokhof et al. [3] (**B**) Program sensitivity in North Rhine-Westphalia estimated based on claims data compared to data published by Bokhof et al. [3]. (both with 95%-confidence intervals)

In our study population of North Rhine-Westphalia, we identified a total of 4,548 incident breast cancers (3,380 screen-detected and 1,168 interval cancers), resulting in a crude rate of 6.5 for screen-detected cancers and a crude rate of 2.2 for interval cancers per 1,000 non-initial screening examinations for the period between 2010 and 2016 overall. Considering each year separately, the age-standardized rate of screen-detected breast cancers varied between 5.4 and 6.5, and the rates of interval cancers varied between 2.0 and 2.3 per 1,000 non-initial screening examinations (Fig. 2A). Based on our data, the program sensitivity for North Rhine-Westphalia ranged between 71.0% and 76.4% between 2010 and 2016 (Fig. 2B). For comparison, Fig. 2 also shows data published by Bokhof et al., who estimated the rate of screen-detected and interval cancer as well as program sensitivity in 2010 and 2011 for North Rhine-Westphalia based on data from the federal state’s cancer registry linked to the data from screening units [3]. The program sensitivity was similar to our estimates (Fig. 2B). The point estimates of the rate of screen-detected and interval cancers were lower than our estimates, but the confidence intervals largely overlapped.

### Overall results and comparison with external data source

**Fig. 3:**
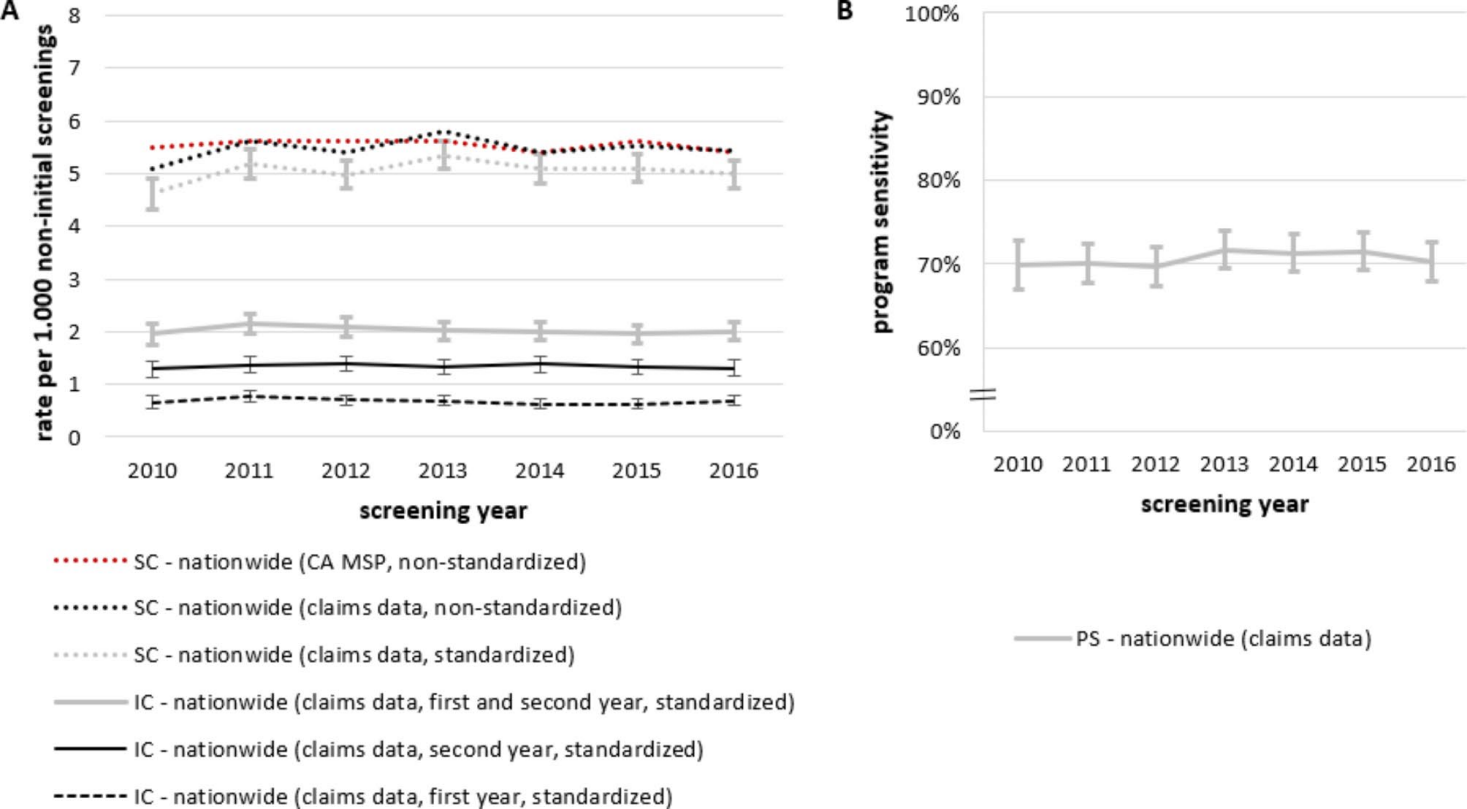
(**A**) Rates of screen-detected (SC) and interval cancer (IC) per 1,000 non-initial screening examinations with 95% confidence intervals between 2010 and 2016. The red line depicts the rates of screen-detected breast cancer as reported by the Cooperative Association (CA) of the German MSP based on data for all non-initial MSP participants [10–16]. Of note, these rates are not age-standardized. To facilitate comparison, we therefore also show the non-standardized rates for claims data, in addition to the age-standardized rates (using the old standard population of Europe as reference). Rates of interval cancers are also shown stratified by the year of diagnosis (first vs. second year after screening attendance). (**B**) Program sensitivity (PS) between 2010 and 2016 with 95% confidence intervals

Overall, we identified a total of 15,074 incident breast cancers (10,916 screen-detected and 4,158 interval cancers), resulting in a crude rate of 5.5 for screen-detected cancers and a rate of 2.2 for interval cancers per 1,000 non-initial screening examinations for the period between 2010 and 2016. Considering each year separately, the age-standardized rate of screen-detected cancer varied between 4.6 in 2010 and 5.3 in 2013 (per 1,000 non-initial screening examinations). Age-standardized rates of interval cancers ranged between 1.9 and 2.1 per 1,000 non-initial screening examinations. Stratification of the rate of interval cancers according to the year when the interval cancer occurred (the first year (quarters 1–4) vs. the second year (quarters 5–8) after screening examination) showed that about two third of interval cancers occurred in the second year (Fig. 3A). As shown in Fig. 3B, the program sensitivity ranged between 69.9% and 71.7%. Figure 3A also depicts the rate of screen-detected cancers as reported by the Cooperative Association of the German MSP, which was 4.8–19.3% higher compared to the rate estimated based on claims data.

## Discussion

To the best of our knowledge, this is the first study investigating the feasibility and plausibility of estimating the rate of screen-detected and interval cancers as well as program sensitivity of the German MSP based on health claims data. Using data until 2016 covering 12% of the German population, we estimated a program sensitivity of about 70–72% for the German MSP, which is well in line with program sensitivity determined for North Rhine-Westphalia by linking data from screening units and cancer registry data [3]. While such a data linkage is resource-intensive and poses several other challenges in Germany, claims data are readily available, i.e. the analyses described here are a useful and pragmatic approach to monitoring quality parameters of the German MSP.

While the interval cancer rate and program sensitivity were reported for North Rhine-Westphalia from the years 2005 to year 2011 and for Lower Saxony from the years 2006 to 2011, there are no other estimates of these parameters for Germany [3]. Accordingly, there is no external data source to which we could compare our more recent estimates. Our results do not indicate that there have been major changes in these parameters over time, but it has to be considered that the precision of our estimates was limited, so detecting minor trends would require an even larger sample size. The Cooperative Association of the German MSP does not have information on interval cancer rates nor on program sensitivity but it annually reports other nationwide (quality) parameters such as the rate of screen-detected breast cancers [10–16]. These rates are slightly higher than the rates of screen-detected cancers observed in our study. A likely explanation for this is that we used rather loose criteria to classify breast cancers as prevalent cases. For example, one outpatient code of breast cancer in the pre-observation period already led to classification as a prevalent case (i.e. this case could no longer become an incident case) and miscoding in the outpatient setting is not very rare. In other words, we may partly have misclassified cancers detected at screening or during follow-up as prevalent cancer and thus may have underestimated the absolute number of screen-detected and interval cancers. As this potential misclassification concerns both screen-detected and interval cancers, we do not think that it has a relevant impact on program sensitivity, which is supported by the good agreement between program sensitivity observed in our study and external data sources.

According to the European guidelines for quality assurance in breast cancer screening and diagnosis, interval cancer rates should not exceed 30% of the background incidence rate in the target population in the months 0–11 after the screening mammography and they should not exceed 50% in the months 12–23 after the screening mammography. The Cooperative Association of the German MSP estimated the background incidence rates of breast cancer using data from the pre-introduction period of the MSP (1999–2005) and cancer registries [17]. For the years 2010 to 2016, the nationwide background incidence was estimated to be 2.6–2.8 cases per 1,000 women. In 2016, for example, the nationwide background incidence was 2.5714 per 1,000 and the rate of IC in 2016 was 0.6956 per 1,000 for the first year in our study, yielding a proportion of 27.05%, i.e. it was below the threshold of 30%; the same applies to the years 2010–2015. Also in the second year after screening the proportion was either below or (with a maximum of 52%) very close to the threshold of 50% (see Supplemental Table 1). However, it has to be taken into account that background incidence rates show regional variation (e.g. 2.98 per 1,000 in Western Germany and 2.18 per 1,000 in Eastern Germany) and should thus ideally be applied on a regional level.

Comparison of our approach to algorithms developed for databases in other countries is difficult given that the type of data, the health care and reimbursement systems (affecting the type of information available) as well as the characteristics of the MSPs differ between countries. Fenton et al. developed and validated an algorithm focusing on the identification of screen-detected breast cancers in Medicare data, so the age range of women differed (≥ 68 as opposed to 50 to 69 in our study) [18]. Furthermore, the algorithm considered only diagnoses and treatments occurring up to one year after a mammogram, which also hampers comparability.

In the interpretation of our study, the following should be taken into account. First, our algorithm identifies screen-detected cases by the occurrence of a pre-operative case conference in combination with an incident diagnosis of breast cancer as defined based on claims data. Prior analyses linking the data from screening units with cancer registries had information on pre-operative histological findings and post-operative cancer verification, so the type of information is not identical. While case conferences are supposed to be held as the last step of diagnostic verification, we cannot rule out that for some MSP participants no case conference is held, which would result in an underestimation of screen-detected cancers. Second, when trying to distinguish incident and prevalent cases in claims data, one has to make compromises [19]. In our study, we prioritized making sure that a case is incident and therefore may have excluded a too large number of prevalent cases. As mentioned before, this may have slightly underestimated the absolute number of screen-detected and interval cancers (and thus also the respective rates) but we do not expect that it had an impact on program sensitivity. Also, the definition of incident breast cancers could be done based on a more sophisticated algorithm (i.e. searching for inpatient diagnosis later during follow-up), but our results do not suggest that misclassification was a major issue. Third, as the underlying populations of different statutory health insurance providers in Germany partly differ e.g. with respect to socioeconomic characteristics and as we did not consider patients with private health insurance (about 10% of the German population), caution is needed when extrapolating the rates observed in our study to the whole of Germany. Our data cover between 12.4 and 14.2% of all German non-initial screenings between 2010 and 2016 [10–16]. To account for differences in the age distribution and facilitate comparison with Bokhof et al. we used age standardization with the old standard population of Europe. The age distribution of this standard population is rather different from the current age distribution in Germany, which has to be considered when interpreting absolute rates. Fourth, incidence rates vary between initial and non-initial MSP participants [20], so it is reasonable to distinguish between both types of participants. Given that identification of initial MSP participants in claims data is subject to uncertainty due to left truncation, we decided to focus on non-initial MSP participations, i.e. women who already had an MSP screening mammography during the pre-observation period. Finally, it should be taken into account that a transfer of individual information on interval cancers from claims data to the screening units is not possible due to data protection regulations. This also means that an evaluation to distinguish interval cancers into overseen breast cancers and de novo breast cancers cannot be done based on our approach.

## Conclusions

In conclusion, our study demonstrates that estimating quality parameters of the German MSP screening with German claims data — i.e. without complex data linkage procedures — is feasible and yields plausible results. It could thus be a valuable additional tool to monitor the German MSP, complementing other activities in this regard. Our study, which is the only one providing more recent estimates of program sensitivity and the rate of interval cancers for the German MSP, suggests that these quality parameters are in line with the European Guidelines. Extension of our approach to other statutory health insurance funds in Germany would be desirable as this would increase the representativeness and precision of nationwide estimates and facilitate analyses on a federal state level.

### Electronic supplementary material

Below is the link to the electronic supplementary material.

Supplementary Material 1

## Data Availability

The data that support the findings of this study are available from the BARMER but restrictions apply to the availability of these data, which were used under license for the current study, and so are not publicly available. Data may however be made available from the author FH upon reasonable request and with permission of the BARMER.

## List of abbreviations

EBM: German uniform assessment standard for medical procedures and services
IC: Interval cancer
ICD-10-GM: International Classification of Diseases, 10th Revision (ICD-10), German Modification
MSP: Mammography screening program
OPS: Operations and Procedures Coding System
PS: Program sensitivity
SC: Screen detected cancer
SHI: Statutory health insurance
