# Supplementary material for: German mammography screening program: program sensitivity between 2010 and 2016 estimated based on German health claims data
Source: BMC Cancer. 2023 Sep 11;23:852. doi: 10.1186/s12885-023-11378-0 (PMC10496211; doi:10.1186/s12885-023-11378-0)
Supplement: Supplementary file 1 — Supplementary Material 1 [file 12885_2023_11378_MOESM1_ESM.docx]

Supplemental Table 1. Interval cancer (IC) proportion and underlying IC rates and nationwide breast cancer (BC) background incidence as reported by the Cooperative Association of the German Mammography Screening Program (18).

| year | nationwide BC background incidence (per 1,000) | first year after screening | | second year after screening | |
| --- | --- | --- | --- | --- | --- |
|  |  | IC rate per 1,000 | IC proportion | IC rate per 1,000 | IC proportion |
| 2010 | 2.6190 | 0.6648 | 25.38% | 1.2845 | 49.05% |
| 2011 | 2.6667 | 0.7710 | 28.91% | 1.3691 | 51.34% |
| 2012 | 2.6667 | 0.6969 | 26.13% | 1.3949 | 52.31% |
| 2013 | 2.6667 | 0.6944 | 26.04% | 1.3283 | 49.81% |
| 2014 | 2.7000 | 0.6297 | 23.32% | 1.3777 | 51.03% |
| 2015 | 2.8000 | 0.6341 | 22.65% | 1.3219 | 47.21% |
| 2016 | 2.5714 | 0.6956 | 27.05% | 1.3131 | 51.06% |
